# Telemedicine Integrated Care Versus In-Person Care Mode for Patients With Short Stature: Comprehensive Comparison of a Retrospective Cohort Study

**DOI:** 10.2196/57814

**Published:** 2024-11-19

**Authors:** Yipei Wang, Pei Zhang, Yan Xing, Huifeng Shi, Yunpu Cui, Yuan Wei, Ke Zhang, Xinxia Wu, Hong Ji, Xuedong Xu, Yanhui Dong, Changxiao Jin

**Affiliations:** 1 Institute of Hospital Management Peking University Third Hospital Beijing China; 2 Information Management and Big Data Center Peking University Third Hospital Beijing China; 3 Department of Paediatrics Peking University Third Hospital Beijing China; 4 Department of Obstetrics and Gynecology Peking University Third Hospital Beijing China; 5 Department of Otolaryngology Peking University Third Hospital Beijing China; 6 Office of Internet Hospital Peking University Third Hospital Beijing China; 7 Department of Medical Affairs Peking University Third Hospital Beijing China; 8 Institute of Child and Adolescent Health School of Public Health Peking University Beijing China; 9 Party Committee Office and Dean’s Office Peking University Third Hospital Beijing China

**Keywords:** telemedicine, telemedicine integrated care mode, short stature, clinical outcomes, health-seeking behaviors, cost analysis, in-person care, mobile health, mHealth, telehealth, eHealth, video virtual visit, access to care, children, pediatrics, China, accessibility, temporal, spatial constraints, chronic disease

## Abstract

**Background:**

Telemedicine has demonstrated efficacy as a supplement to traditional in-person care when treating certain diseases. Nevertheless, more investigation is needed to comprehensively assess its potential as an alternative to in-person care and its influence on access to care. The successful treatment of short stature relies on timely and regular intervention, particularly in rural and economically disadvantaged regions where the disease is more prevalent.

**Objective:**

This study evaluated the clinical outcomes, health-seeking behaviors, and cost of telemedicine integrated into care for children with short stature in China.

**Methods:**

Our study involved 1241 individuals diagnosed with short stature at the pediatric outpatient clinic of Peking University Third Hospital between 2012 and 2023. Patients were divided into in-person care (IPC; 1183 patients receiving only in-person care) and telemedicine integrated care (TIC; 58 patients receiving both in-person and virtual care) groups. For both groups, the initial 71.43% (average of 58 percentages, with each percentage representing the ratio of patients in the treatment group) of visits were categorized into the pretelemedicine phase. We used propensity score matching to select individuals with similar baseline conditions. We used 7 variables such as age, gender, and medical insurance for the 1:5 closest neighbor match. Eventually, 115 patients in the IPC group and 54 patients in the TIC group were selected. The primary clinical outcome was the change in the standard height percentage. Health-seeking behavior was described by visit intervals in the pre- and post-telemedicine phases. The cost analysis compared costs both between different groups and between different visit modalities of the TIC group in the post-telemedicine phase.

**Results:**

In terms of clinical effectiveness, we demonstrated that the increase in height among the TIC group (Δ*z*_TIC_=0.74) was more substantial than that for the IPC group (Δ*z*_IPC_=0.51, *P*=.01; paired *t* test), while no unfavorable changes in other endpoints such as BMI or insulin-like growth factor 1 (IGF-1) levels were observed. As for health-seeking behaviors, the results showed that, during the post-telemedicine phase, the IPC group had a visit interval of 71.08 (IQR 50.75-90.73) days, significantly longer than the prior period (51.25 [IQR 34.75-82.00] days, *P*<.001; *U* test), whereas the TIC group’s visit interval remained unchanged. As for the cost per visit, there was no difference in the average cost per visit between the 2 groups nor between the pre- and post-telemedicine phases. During the post-telemedicine phase, within the TIC group, in-person visits had a higher average total cost, elevated medical and labor expenses, and greater medical cost compared with virtual visits.

**Conclusions:**

We contend that the rise in medical visits facilitated by integrating telemedicine into care effectively restored the previously constrained number of medical visits to their usual levels, without increasing costs. Our research underscores that administering prompt treatment may enable physicians to seize a crucial treatment opportunity for children with short stature, thus attaining superior results.

## Introduction

From the original transmission of electrocardiograms to the current use of asynchronous and synchronous audio and video virtual visits, telemedicine has become a service approach that provide patients with prevention, diagnosis, treatment, and continuing education [1-3]. Since the successful implementation of the Space Technology Applied to Rural Papago Advanced Health Care project, telemedicine has demonstrated its value and potential as a useful tool for physicians to provide quality health care to people in a remote area [4]. Integrating telemedicine into care has demonstrated equivalent or superior clinical outcomes to traditional in-person care when treating certain diseases, including diabetes [5,6] and hypertension [7,8]. However, in numerous studies, telemedicine was mostly used as a supplementary tool for conventional care, serving as a platform for patient-physician interactions and a channel for patient education [9-12]. To address the need for health care in remote areas, further investigation is required to determine the therapeutic efficacy of telemedicine-enabled virtual visits as a substitute for in-person visits.

Telemedicine not only delivers clinical effectiveness but also enhances accessibility. Telemedicine transcends temporal and spatial constraints [13], mitigating the risk of delayed or missed medical consultations for patients with chronic diseases [8,14,15]. Consequently, it curtails the financial burden associated with untimely interventions [16,17]. The assessment of access mostly relies on the quantification of medical consultations (ie, number of visits in a given period). Research on telemedicine’s impact on improving access can be categorized into 2 distinct groups. The first type of research centered around telemedicine shows that it helps increase health care delivery for patients living in remote areas [18,19]. Another stream of study is based on the premise that the lockdown resulting from the COVID-19 pandemic led to a decrease in in-person outpatient appointments, which was partially compensated by the utilization of telemedicine [20-22]. Although the limits on medical resources resulting from COVID-19 have been alleviated, people in remote and underdeveloped regions continue to grapple with the issue of inadequate medical resources. The significance of telemedicine’s contribution to enhancing accessibility thus remains a subject of importance.

Short stature is a medical term used to describe a condition in which a person’s height is at least 2 SD below the average height for their age, sex, and population group [23]. Height is a personal attribute that is determined by a combination of hereditary and environmental variables [24]. The prevalence of short stature in children ranges from 0.5% to 11.3% [23]. Although the prevalence varies depending on country, people, and gender, studies have revealed that rural areas exhibit a higher prevalence than urban areas, while less economically developed regions have a higher prevalence than more economically developed regions [25]. In these rural or lower-income regions, monetary resources alone are insufficient: Using technological methods to modify the service model is critical to address the issue of short stature [26]. Intervention has a specific time frame within which it is most effective; hence, prompt and consistent intervention might improve outcomes [27]. Furthermore, short stature is listed as one of the main reasons for pediatric consultations [28]. Hence, comprehensive comparison of telemedicine integrated into care with in-person care for children with short stature provides an example for future technological interventions and impact evaluations in remote and underprivileged regions.

**Figure 1:**
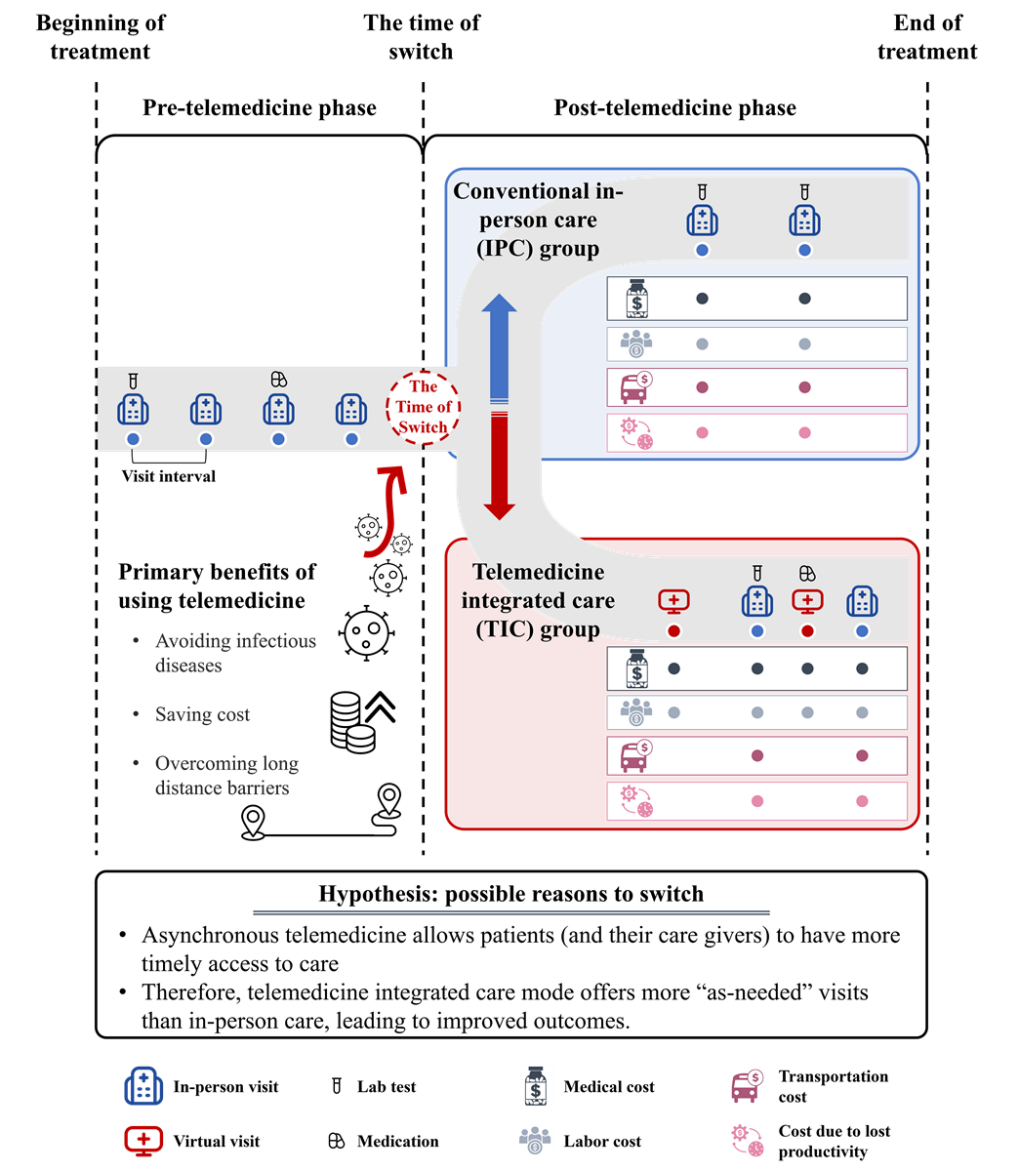
Illustration of conventional in-person and telemedicine integrated care modes.

Currently, there is a lack of evaluation of telemedicine considering both clinical outcomes and access to care. We hypothesized that telemedicine improves accessibility by allowing patients to receive more timely visits than in-person care, leading to improved outcomes (Figure 1). To investigate this theory, we used short stature as an example and conducted a retrospective cohort study including patients who only had in-person visits (in-person care [IPC] group), and patients who had both virtual and in-person visits (telemedicine integrated care [TIC] group), which started to occur when certain individuals pursued virtual care because of concerns such as avoiding infection [29]. We performed a comprehensive comparison between the 2 cohorts, examining clinical outcomes, health-seeking behaviors, and average cost per visit associated with the 2 treatment modalities. The “time of switch” indicates the ratio of the number of visits related to the first virtual visit to the total number of visits in the TIC group. Each in-person visit involves 4 categories of costs: medical, labor, transportation, and loss of productivity. Every virtual visit only involves the first 2 categories of costs.

## Methods

### Telemedicine Integrated Care Mode

The Department of Paediatrics at Peking University Third Hospital provides comprehensive services for patients with short stature, including both in-person and virtual visits. The virtual visits are done asynchronously, meaning the physicians respond to the patients’ inquiries at their own convenience [30]. Similar to in-person appointments, virtual visits enable physicians to prescribe medications and request laboratory tests for patients. After the initial in-person visit with a pediatrician, patients have the option to either continue with conventional in-person visits or transition to the integrated telemedicine mode (Figure 1). However, with regards to medical insurance reimbursement, the reimbursement policy permits online direct settlement exclusively for medical service fees while excluding prescription fees and charges related to inspections and testing. Patients who choose virtual visits are required to bear the financial responsibility for any medication or test fees incurred should they wish to make online payments for such expenses. In order to mitigate this phenomenon, we used the patient’s specific medical insurance coverage as a variable when assessing its potential impact on health-seeking behaviors.

### Study Design and Data Collection

Our study focused on patients who underwent therapy for short stature in the pediatrics outpatient department at Peking University Third Hospital from March 1, 2012, to March 31, 2023. The implementation of the electronic medical record (EMR) system occurred in the year 2012. Prior to that, the storage of medical records relied solely on paper-based systems, which posed difficulties for accessing precise and comprehensive medical information. The study did not include patients who had visit records for short stature between April 2023 and December 2023, as these recent visit records indicated incomplete treatment cycles. Patients under screening were divided into 2 cohorts based on their visit modalities. Patients who had only in-person visits were in the IPC group, and those who had both virtual and in-person care were in the TIC group (Figure 1).

During each appointment, demographic information such as age, gender, and insurance type, as well as behavioral data related to the visit, including the date, type (in-person or virtual), and diagnosis, were collected using the hospital information system. The initial occurrence of a diagnosis indicating short stature was designated as the date of diagnosis. Simultaneously, the age of the patient at the time of diagnosis was denoted as the age of diagnosis. The treatment duration was operationally defined as the temporal interval between the date of the patient’s most recent diagnosis of short stature and the date of diagnosis. The visit interval was defined as the number of days between 2 consecutive visits. The mean visit interval for each individual patient was calculated, followed by computing the average interval across all patients within each group. Every patient was given equal weight, regardless of the number of visit intervals they had. Clinical outcomes, including height and levels of insulin-like growth factor 1 (IGF-1) and 25-hydroxyvitamin D (25-OH VitD3), were retrieved from the EMR to assess clinical effectiveness.

The cost data encompassed medical expenses, labor expenditures, transportation fees, and costs associated with lost production. The medical expenses associated with each visit were obtained from the EMR system. The labor cost was determined by multiplying the average physician compensation per unit of time by the average service time. The salary was derived by comparing the incomes of physicians in 3 different positions: chief physician, deputy chief physician, and attending physician. The hospital financial department submitted the data. The average service time for each in-person visit was calculated by dividing the total time spent in one outpatient unit by the average number of patient visits per outpatient unit. The outpatient unit had a length of 4 hours. In order to ascertain the length of virtual visits, which posed difficulties for measurement due to their asynchronous character, we conducted interviews with 19 physicians, who accounted for 95% (19/20) of the entire population in the Department of Paediatrics that took part in virtual visits. The calculation of transportation expenses relied on the patients’ location data. The cost of a single taxi ride spanning a distance of 10 kilometers for patients living in Beijing was calculated as ¥30 (US $4.18) [31], leading to a total transportation cost of ¥60 (US $8.36) for each in-person visit. To calculate the cost for patients who lived outside of Beijing, we determined the transportation cost by considering the least expensive price for a second-class train ticket from their location to Beijing. The cost of lost productivity was calculated based on the mean salary of urban workers in Beijing during the year 2019 [32]. It was assumed that the duration of in-person visits was 4 hours and 8 hours for patients residing in Beijing and in other cities, respectively. Engaging in virtual visits did not result in any costs related to lost productivity.

### Propensity Score Matching (PSM)

Of the total sample size of 1241 individuals, 1183 participants opted to proceed with in-person visits, constituting the IPC group. Conversely, 58 individuals chose to explore telemedicine as an alternative, thereby forming the TIC group. The 2 groups underwent propensity score matching (PSM) to mitigate the potential influence of unobserved confounding variables. The matching and quality assessment were conducted using the *psmatch* and *pstest* functions in Stata.

For the purpose of matching, various factors like age at diagnosis, date of diagnosis, gender, and insurance type were considered as covariances. The 2 most critical variables in characterizing patient health-seeking behavior, namely visit intervals in the pre-telemedicine phase and treatment duration, were also chosen as criteria for the PSM. The 1:5 closest neighbor with replacement technique was used for its ability to provide high-quality matches, with biases of less than 10%, across all 6 attributes (Figure 1).

### Statistical Analysis

The paired *t* test was used to examine the presence of significant alterations in the standard height percentage value (*z* score) associated with the height and BMI of patients within each cohort before and after the treatment (*z* score). An independent sample *t* test was used to examine if there was a significant difference in *z* scores of height and BMI between the 2 groups. The analyses were conducted using the Stata command *ttest.* The reference ranges for IGF-1, free thyroxine (FT4), and thyroid-stimulating hormone (TSH) were used to determine normal values. By identifying individuals whose measurements fell within these normal ranges, the Stata command *ranksum* was used to assess the consistency between the 2 groups in the pre- and post-telemedicine phases. The Wilcoxon rank sum test was also used to assess if there was a statistically significant difference in 25-OH VitD3 concentration between the 2 groups. Because the distribution of visit intervals and cost did not conform to a normal distribution, the median and IQR of visit intervals and cost per visit in both the IPC and TIC groups were analyzed using the Wilcoxon rank sum test with the Stata command *ranksum*.

### Ethical Considerations

This study conducted a secondary analysis of existing research data, for which informed consent was obtained during the original data collection. Consequently, the Peking University Third Hospital Medical Science Research Ethics Committee permitted this secondary analysis without requiring additional patient consent. The IRB approval number is IRB00006761-M2023394.

All research data involving human subjects in this study have been anonymized to safeguard participant privacy and data security. This research did not offer any compensation to the participants.

## Results

### Cohort Characteristics

**Figure 2:**
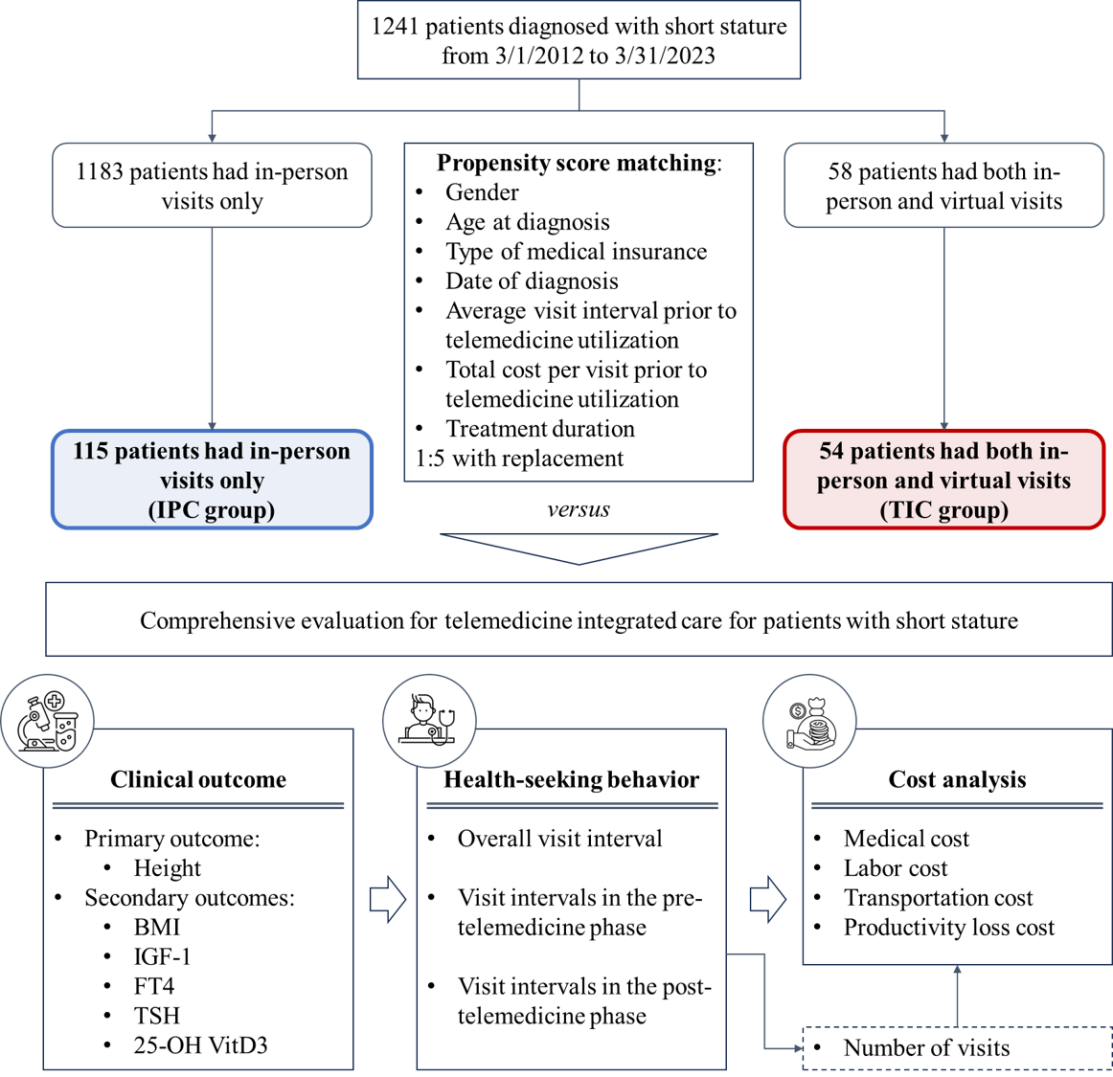
Study flow diagram. 25-OH VitD3: 25-hydroxyvitamin D3; FT4: free thyroxine; IGF-1: insulin-like growth factor 1; TSH: thyroid stimulating hormone.

From March 2012 to March 2023, a total of 1241 patients presenting with short stature sought medical treatment at the Department of Paediatrics in Peking University Third Hospital. The majority of the 1241 participants in the study opted for in-person visits with pediatricians (n=1183), whereas a smaller subset of 58 participants decided to use telemedicine at some point in the treatment (Figure 2).

The 2 patient groups were comparable in age at diagnosis, date of diagnosis, composition of medical insurance types, visits intervals, and total costs prior to the adoption of care integrating telemedicine by the TIC group. However, there was a statistically significant higher proportion of female patients in the TIC group (*P*=.03; chi-square). These patients also exhibited a longer median treatment length of 650 (IQR 508-1144) days, as compared with a median treatment duration of 434 (IQR 224-749) days for patients who had in-person visits only (*P*<.001; *U* test; Table 1).

**Table 1:** A summary of the patients’ demographic information.

Characteristics			Total sample					*P* value				PSM^a^ subsample						*P* value	
			IPC^b^ group (n=1183)		TIC^c^ (n=58)						IPC group (n=115)				TIC group (n=54)				
						
Female, n (%)			581 (49.1)		37 (63.8)		.03				71 (61.7)				34 (63)		.88		
Age at diagnosis (years), median (IQR)			10 (8-12)		10 (8-11)		.11				10 (7-11)				10 (8-11)		.74		
**Medical insurance, n (%)**									.22										.12
	Beijing Urban and Rural Medical Insurance	990 (83.7)		52 (89.7)						111 (96.5)				49 (90.7)					
	Out of pocket	189 (16)		6 (10.3)						4 (3.5)				5 (9.3)					
	Cross-provincial medical insurance	4 (0.3)		0						0				0					
**Date of diagnosis, n (%)**									.25										.38
	2013-2017	61 (5.2)		5 (8.6)						11 (9.6)				3 (5.6)					
	2018-2022	1122 (94.8)		53 (91.4)						104 (90.4)				51 (94.4)					
Average visit interval in the pretelemedicine phase (days), median (IQR)			52.50 (33.80-83.89)		56.95 (36.16-76.73)		.60				51.25 (34.75-82.00)				58.13 (36.32-77.45)		.59		
Treatment duration (days), median (IQR)			434 (224-749)		650 (508-1144)		<.001				652 (434-1280)				650 (508-1070)		.59		
Total cost per visit in the pretelemedicine phase (¥)^d^, median (IQR)			3541.93 (2308.50-5704.29)		3856.62 (2618.48-5698.33)		.23				3098.99 (2176.71-5266.23)				3412.62 (2907.62-4705.71)		.10		

^a^PSM: propensity score matching.

^b^IPC: in-person care only.

^c^TIC: telemedicine integrated care, including both in-person and virtual care.

^d^A currency exchange rate of ¥1=US $0.14 is applicable.

### The Time of Switch

To examine the impact of care integrating telemedicine, it is important to select patients who had comparable characteristics before the use of telemedicine. A notion called “the time of switch” was developed to divide the treatment course into pre-telemedicine and post-telemedicine phases. The time of switch refers to the ratio of the number of visits related to the first virtual visit to the total number of visits by the TIC group. In our calculation, the time of switch was 71.43% (average of 58 percentages, with each percentage representing the ratio of patients in the treatment group). As for those who only had in-person care, due to the absence of telemedicine use, we allocated the same proportion of visits as observed for the patients using both types of visits to the patients who only had in-person care. Specifically, we assigned the initial 71.43% of visits to the pretelemedicine phase and the rest to the post-telemedicine phase. During this phase, a total of 107 virtual visits were recorded. Of these, 44 patients (81.5%) subsequently had in-person visits. It is worth noting that only 1 patient had both virtual and in-person visits on the same day (Figure 1).

### PSM

Following the establishment of pre- and post-telemedicine phases in both groups, we used PSM to select patients with similar baseline conditions to evaluate the clinical outcomes, change in health-seeking behaviors, and related costs. The matching process was based on 7 initial parameters in the pre-telemedicine phase, including gender, age at diagnosis, type of medical insurance, date of diagnosis, visit interval, treatment duration, and total cost per visit. A total of 54 patients constituted the TIC group and were paired with 115 patients in the IPC group. The 2 matched cohorts exhibited no statistical differences in all 7 factors; hence, we considered this matching process acceptable. The matched groups were largely female patients with Beijing Urban Residents Medical Insurance who were diagnosed at the age of 10 years during 2018 to 2022. The treatment usually lasts for about 650 days, during which the patients had in-person visits once every 51 or 58 days, with a total cost of over ¥3000 (US $471.82) per visit (Table 1). These patients were included for further analysis of clinical outcomes, health-seeking behaviors, and costs.

### Clinical Outcomes

#### Alteration in Height

**Figure 3:**
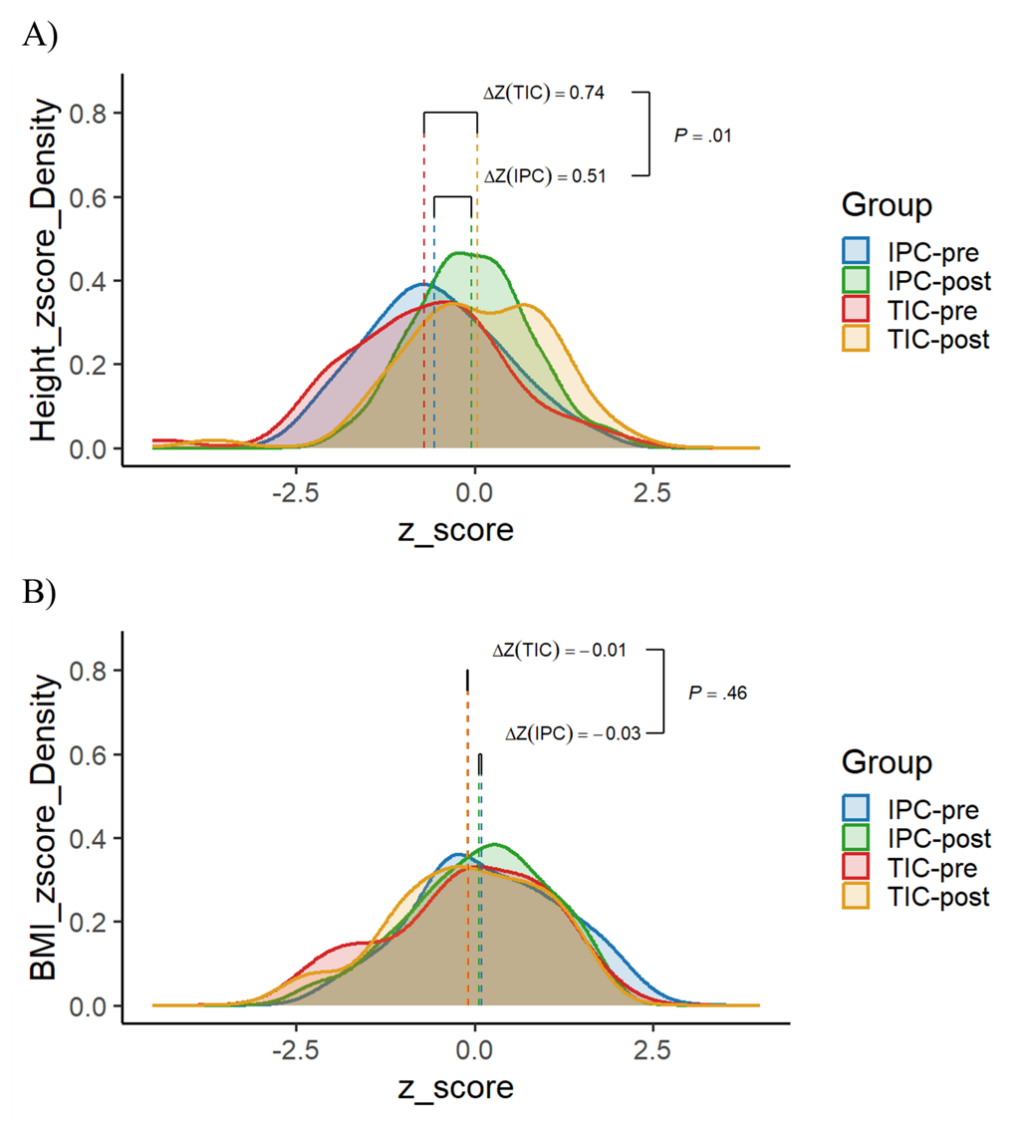
Pre- and post-telemedicine z score distributions of (A) height and (B) BMI, with the dashed lines representing the mean value of the z scores for each cohort. IPC: in-patient care only; TIC: telemedicine integrated care.

The primary outcome to assess the impact of interventions targeting short stature is the measurement of height. Height was measured at the beginning and end of treatment using the standard height percentage value, or *z* score, established by the World Health Organization (WHO) for children aged 0 years to 18 years. The height *z* scores for patients improved from –0.556 to –0.044 in the IPC group and –0.698 to 0.047 in the TIC group. The *z* score in both groups exhibited a statistically significant increase, yet the improvement for patients in the TIC group was more pronounced (*P*=.01; paired *t* test; Figure 3A).

#### Alteration in BMI

Comparing BMI with the appropriate age- and sex-specific growth chart enables pediatric health care providers to monitor growth and identify potential health- or nutrition-related problems. Similar to height, BMI was measured at the beginning and end of treatment and evaluated according to WHO standards. As the height of both groups experienced a substantial increase, the BMI *z* scores of both groups exhibited a decrease. Specifically, the *z* score of the IPC group decreased by 0.03, while that of the TIC group decreased by 0.01. However, it is worth noting that the BMI of both cohorts remained within the normal range and there was no significant disparity in the extent of decrease (Figure 3B).

#### Alterations in Other Clinical Indicators

##### Change in IGF-1

**Figure 4:**
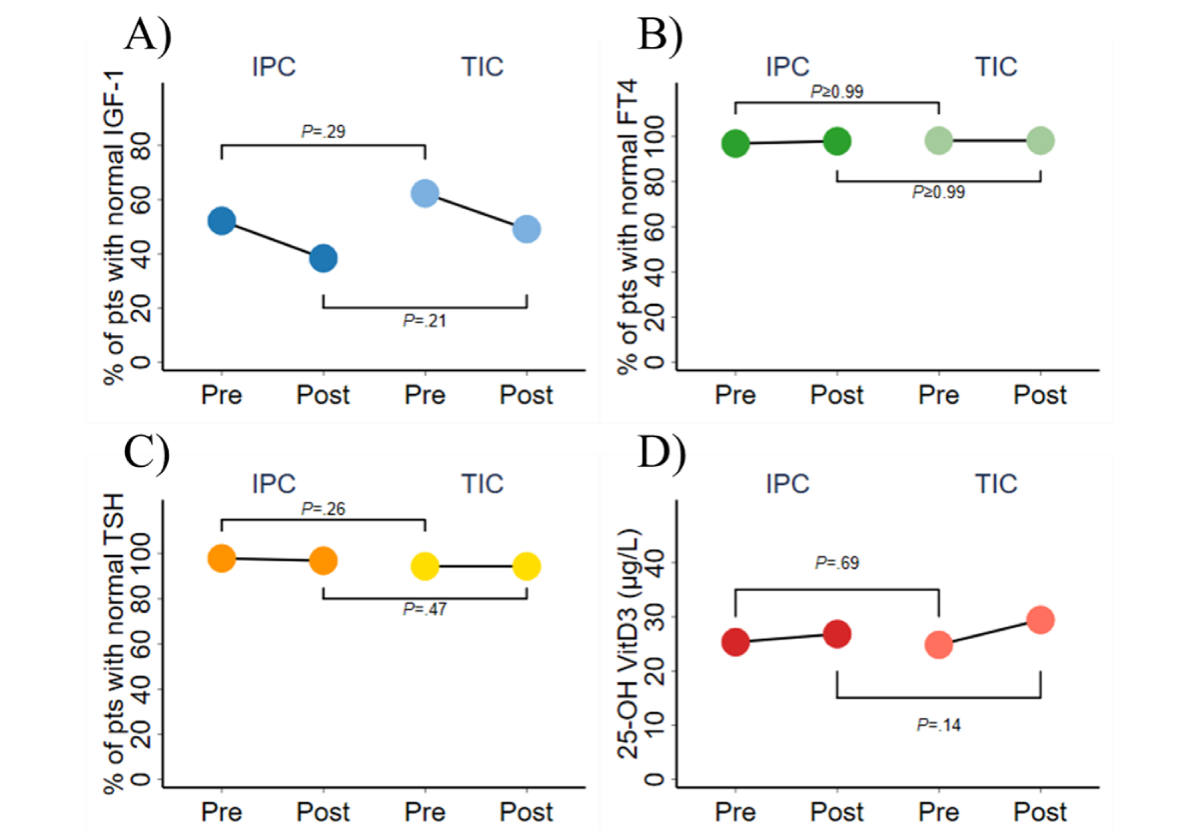
Changes in the secondary clinical outcomes between pre-telemedicine and post-telemedicine: (A) insulin-like growth factor 1 (IGF-1; normal range: 64-345 ng/mol), (B) free thyroxine (FT4; normal range: 0.89-1.8 pmol/L), thyroid-stimulating hormone (TSH; normal range: 0.65-6.27 pmol/L), (D) 25-hydroxyvitamin D3 (25-OH VitD3; ≥20 μg/L). pts: patients.

IGF-1 is important for the processes of skeletal and general growth and development. Consequently, it is frequently investigated in the context of therapeutic interventions for individuals with short stature. This study observed a high percentage (53/54, 98%) of patients using care integrating telemedicine who had their IGF-1 levels measured both at the outset and toward the end of their treatment. At the beginning of treatment, the majority of patients (IPC group: 50/94, 53%; TIC group: 33/53, 62%) had normal IGF-1 levels. However, subsequent analysis revealed a decrease in this proportion, with the IPC group experiencing a reduction to 38% (36/94) and the TIC group experiencing a reduction to 49% (26/54). Nevertheless, there was no statistically significant difference observed between the 2 groups in any of the parameters. In both groups, the combined measurements accounted for about 95% of the length of treatment (IPC: median 92.4%; TIC: median 97.2%), thus representing the entirety of the treatment process well (Figure 4A).

##### Change in FT4 and TSH

The thyroid gland is a vital endocrine gland responsible for the secretion of thyroid hormones. Thyroid hormones play a crucial role in facilitating growth and development and maintaining skeletal health. A significantly higher number of patients in the TIC group possessed comprehensive records of FT4 and TSH measurements compared with those in the IPC group. Throughout the treatment course, the percentage of patients with FT4 values within the normal range in both groups did not significantly change. Furthermore, there was no statistically significant difference between the 2 groups. Both groups exhibited over 90% of the total duration between the 2 assessments (IPC: median 91.9%; TIC: median 95.5%), effectively capturing the entirety of the treatment course (Figures 4B and 4C).

##### Change in 25-OH VitD3

Vitamin D has a crucial role in the processes of calcium metabolism and bone growth. The predominant form of vitamin D kept in blood is 25-OH VitD3. By the conclusion of the treatment, both groups exhibited an increase in 25-OH VitD3 levels. The concentration in the IPC group increased from a median 25.30 (IQR 17.20-30.10) μg/L to 26.80 (IQR 22.80-34.40) μg/L, whereas the concentration in the TIC group increased from a median 24.80 (IQR 18.70-31.01) μg/L to 29.40 (IQR 25.30-35.60) μg/L. However, there was no statistically significant difference between the TIC group and IPC group at either time point. In both cohorts, the 2 measurements collectively represented more than 90% of the treatment time (IPC: median 92.7%; TIC: median 96.2%), encompassing a significant portion of the entire treatment duration (Figure 4D).

### Health-Seeking Behaviors

#### Visit Intervals

**Figure 5:**
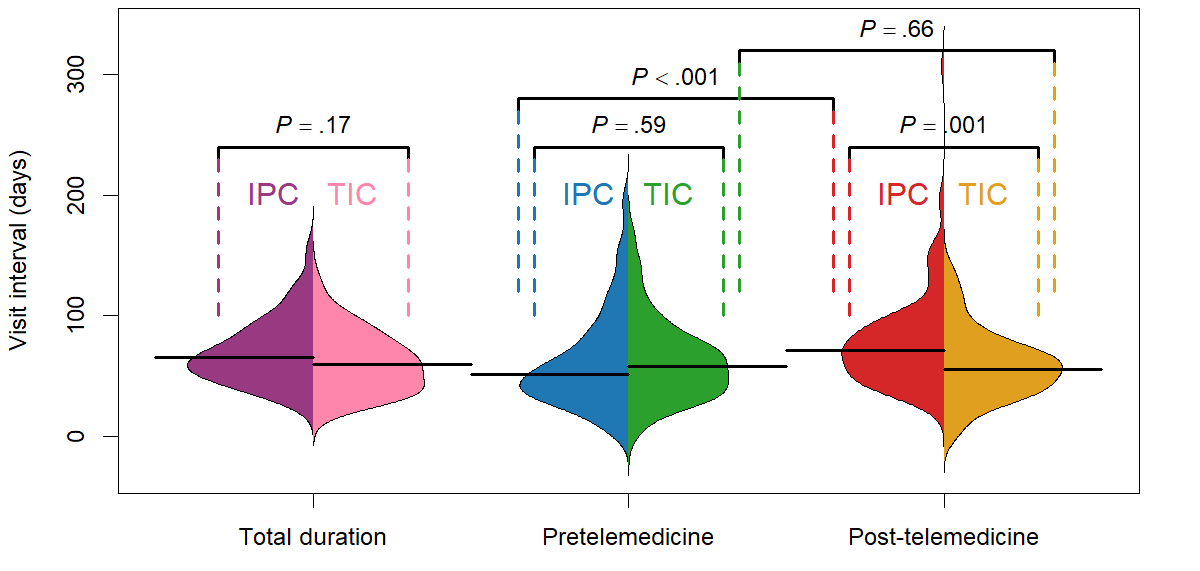
Bean plots of the distribution of the visit intervals for the in-person care (IPC) group and telemedicine integrated care (TIC) group, with differences assessed using Wilcoxon rank sum tests and the solid black lines representing the median value of the variable being analyzed.

The median visit interval for the IPC group was 65.20 (IQR 51.50-85.67) days. For the TIC group, the median visit interval was 61.04 (IQR 45.08-76.11) days. There was no significant difference in the visit intervals between the 2 groups (*P*=.17; *U* test; Figure 5).

#### Visit Intervals in the Pretelemedicine Phase

In this phase, the IPC and TIC groups had intervals between visits of 51.25 (IQR 34.75-82.00) days and 58.13 (IQR 36.32-77.45) days, respectively. There was no statistically significant difference between the 2 groups (*P*=.59; *U* test; Figure 5).

#### Visit Intervals in the Post-Telemedicine Phase

After adopting care integrating telemedicine, the TIC group had average visit intervals of 55.54 (IQR 38.86-70.25) days, which was comparable to that in the preceding phase. Nevertheless, the IPC group had a substantial increase in the median average interval, amounting to 71.08 (IQR 50.75-90.73) days (*P*<.001; *U* test), which was also significantly longer than that for the TIC group (*P*=.001; *U* test; Figure 5).

### Cost Analysis

#### Total Cost Per Visit

**Figure 6:**
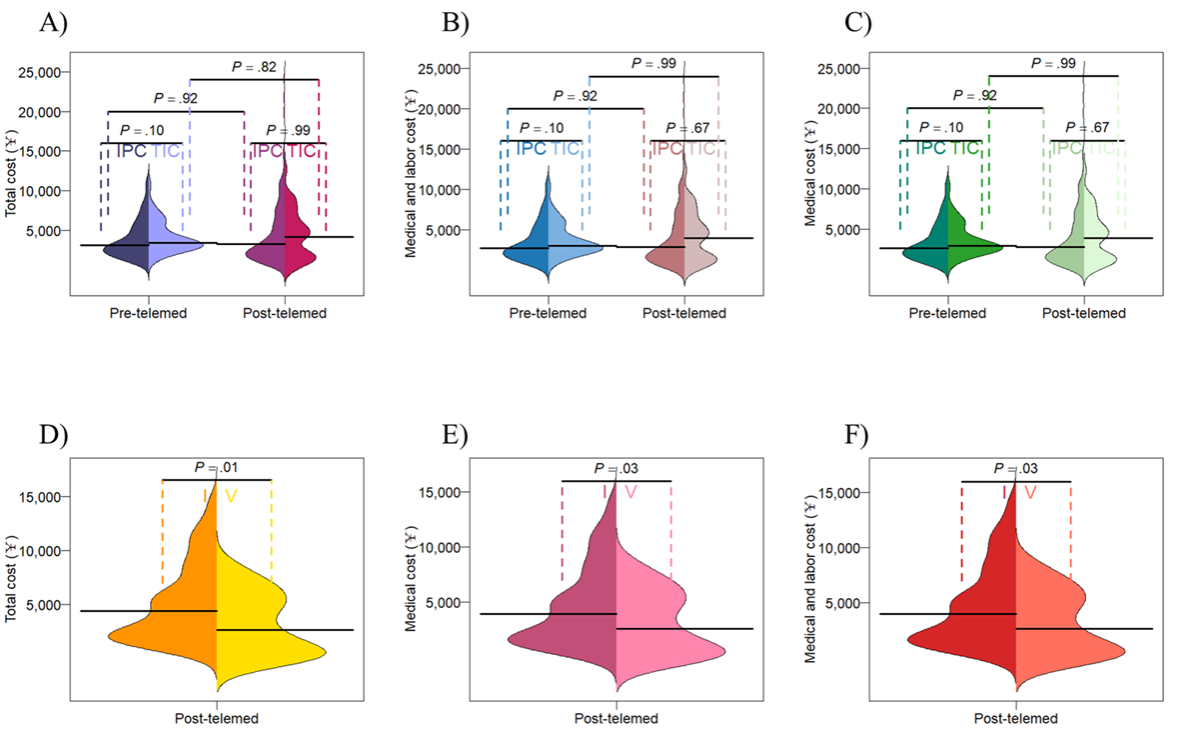
Bean plots showing the costs per visit: (A) total cost per visit compared between the in-person care (IPC) and telemedicine integrated care (TIC) groups in the pre- and post-telemedicine phases, (B) medical and labor costs per visit compared between the IPC and TIC groups in the pre- and post-telemedicine phases, (C) medical costs per visit compared between the IPC and TIC groups in the pre- and post-telemedicine phases, (D) total cost per visit for the TIC group compared between the in-person (I) and virtual (V) visits, (E) medical and labor costs per visit compared between the I and V visits, (F) medical costs per visit compared between the I and V visits, with differences assessed using Wilcoxon rank sum tests and the solid black lines representing the median value of the variable being analyzed.

The total cost consists of medical costs, labor costs, transportation costs, and lost productivity costs. In the pre-telemedicine phase, the median total cost per visit was ¥3089.99 (IQR 2176.71-5266.23; US $430.35 [IQR 303.16-655.37]) and ¥3412.62 (IQR 2907.62-4705.71; US $475.28 [IQR 404.95-655.37]) in the IPC and TIC groups, respectively. There was no significant disparity in the total cost per visit between the 2 cohorts. In the post-telemedicine phase, the median total costs per visit were ¥3239.73 (IQR 1808.45-6331.82; US $451.20 [IQR 251.87-881.85]) and ¥4169.14 (IQR 1726.21-6042.05; US $580.64 [IQR 240.41-841.49]) in the IPC and TIC groups, respectively. The average total cost per visit was not statistically different between the 2 groups within each phase nor before and after the use of telemedicine within each group (Figure 6A).

#### Medical and Labor Costs Per Visit

Although virtual consultations eliminate expenses such as costs of transportation and lost productivity, there are still direct costs to consider, such as medical and labor costs. In the pretelemedicine phase, the medical and labor costs were ¥2692.58 (IQR 1770.30-4859.82; US $375.00 [IQR 246.55-676.84]) for the IPC group and ¥3006.21 (IQR 2501.21-4299.30; US $418.68 [IQR 348.35-598.77]) for the TIC group. During the post-telemedicine phase, the medical and labor costs for the 2 groups amounted to ¥2833.32 (IQR 1402.04-5925.41; US $394.60 [IQR 195.27-825.24]) and ¥3923.60 (IQR 1455.27-5798.21; US $546.45 [IQR 202.68-807.53]), respectively. There were no discernible changes for each group between the pre- and post-telemedicine phases nor any differences between the 2 groups within the same phase (Figure 6B).

#### Medical Cost Per Visit

Typically, pediatricians allot an average of 7.84 minutes for in-person visits, and they dedicate approximately 10 minutes for each virtual visit, resulting in an average of ¥12.46 (US $1.74) per visit in labor costs due to different treatment modalities. To specifically assess the influence of telemedicine on costs, we performed another analysis focusing solely on medical costs, which included prescriptions, laboratory tests, and registration fees. In the pretelemedicine phase, the medical costs were ¥2647.34 (IQR 1725.06-4814.59; US $368.70 [IQR 240.25-670.54]) for the IPC group and ¥2960.97 (IQR 2455.97-4254.06; US $412.38 [IQR 342.05-592.47]) for the TIC group. During the post-telemedicine phase, the medical costs for the 2 groups were ¥2788.09 (IQR 1356.80-5880.18; US $388.30 [IQR 188.96-818.94]) and ¥3873.43 (IQR 1405.88-5747.99; US $539.46 [IQR 195.80-800.53]), respectively. There were no discernible changes for each group between the pre- and post-telemedicine phases nor any differences between the 2 groups within the same phase (Figure 6C).

#### Cost Per Visit in the Post-Telemedicine Phase for the TIC Group

To facilitate a more natural comparison of costs across different medical visit channels, we conducted an analysis of various costs incurred by types of visits for the same group of patients at the same phase. Our findings revealed that, in the post-telemedicine phase, the total cost, medical and labor costs, and medical costs associated with the in-person visits were all much greater than these costs generated by virtual visits in the TIC group (Figures 6D, 6E, and 6F).

## Discussion

### Principal Findings

We conducted a rigorously planned retrospective cohort study to thoroughly examine the disparities in clinical outcomes, health-seeking behaviors, and average expenses per visit between care integrating telemedicine and in-person care only using short stature as an example. Our analysis revealed that the short stature patients in the TIC group had more significant growth than the IPC group. Meanwhile, this novel approach did not result in any negative impacts on other metrics such as BMI and IGF-1 level. Furthermore, our observations demonstrated that the patients in the TIC group consistently adhered to the same visit frequency throughout the whole treatment cycle, whereas patients in the IPC group prolonged their visits by 38.68% of the time during the post-telemedicine phase. Last, we observed that the average cost per visit in the TIC group was indistinguishable from that of the IPC group, yet the average cost per visit of virtual visits was cheaper than that of the usual in-person visits. By conducting a thorough investigation of 2 service models, we successfully demonstrated the effective implementation of telemedicine in China. This serves as compelling proof for the use of telemedicine in clinical treatment.

Telemedicine is used in domains such as diabetes [5,6] and hypertension [7,8]. These therapeutic domains have 3 attributes in common. First, all necessitate regular and consistent checkups over an extended period [33]. Second, access to care has an impact on the results [34,35]. Third, treatment is subject to a specific timeframe, meaning the timelier the intervention, the more pronounced the impact [36]. The characteristics of short stature in children align with these criteria; however, there is a lack of research examining the potential impact of telemedicine on improving results in this domain. We addressed this deficiency in our research. Given that children in lower-income regions have a higher prevalence of short stature [25], we considered not only the clinical efficacy but also the patients’ health-seeking behaviors and financial burden to comprehensively evaluate the feasibility of implementing telemedicine in the perspective of value-based medicine [37].

Our research on health-seeking behaviors has shown a novel finding: There were variations in the visit intervals depending on the different modalities of treatment. This finding strongly supports the notion that telemedicine can improve clinical outcomes by enhancing accessibility. By comparing the visit intervals between the 2 groups in the post-telemedicine phase, we conclude that telemedicine enhanced accessibility. The 2 propensity score–matched cohorts should exhibit the same health-seeking behaviors, yet in the post-telemedicine phase, the visit intervals for patients in the TIC group remained the same as in the previous phase, whereas patients receiving in-person care had prolonged intervals. This indicates that telemedicine is a more efficient means of ensuring regular visits, making it a valuable instrument in situations when medical availability is restricted. For instance, during the pandemic, patients deferred or canceled their scheduled appointments due to concerns regarding coronavirus infection [38,39]. Telemedicine thereby demonstrated its benefits of allowing patients to receive health care service from afar, making it easier to adhere to the treatment schedule [40,41]. By comparing the visit intervals in the pre- and post-telemedicine phases in the TIC group, we deduced that telemedicine did not promote unnecessary medical visits. Since the visit intervals of the TIC group remained the same across the 2 phases, it is believed that the visit frequency stayed consistent. This suggests that the use of telemedicine did not result in a reduction of the interval nor did it result in excessive use of medical resources, as some researchers have expressed concern about [42]. Additionally, the clinical findings illustrate that the growth in height in the TIC group was more significant, while no statistically significant difference was observed in other clinical parameters between the 2 groups. This demonstrates that the efficacy of integrating telemedicine into care was superior and did not yield any unfavorable effects. Regarding the conclusion that telemedicine “triggered” a higher number of visits, our findings align with the research conducted by Bavafa et al [43]. Another conclusion that telemedicine did not reduce the visit interval also aligns with previously reported results [44]. Nevertheless, our work builds upon prior research and aims to comprehensively examine the mechanism of telemedicine effectiveness by integrating clinical efficacy with health-seeking behaviors. In conclusion, we contend that, during the post-telemedicine phase, the rise in medical visits facilitated by integrating telemedicine into care effectively restored the previously constrained number of medical visits to their usual levels. Our results suggest that telemedicine enhances the efficacy of short stature treatment by enhancing accessibility of care.

Our study also examined the costs associated with different modalities in the new application to short stature. We found that the average cost per visit of patients revealed no significant difference between the 2 groups or within the pre- and post-telemedicine phases. The average cost was calculated based on the patient’s cohort affiliation, meaning it encompassed the cost associated with both in-person visits and virtual visits. The comparable average cost per visit suggests homogeneity of the 2 groups. Subsequently, we consider virtual sessions as a replacement for in-person visits, rather than as a supplementary method like patient education or a social network support [9-12], in order to delve deeper into the correlation between visit modalities and costs. The analysis revealed that the average total cost per visit was lower for virtual visits. Telemedicine has the potential to reduce the total cost per visit, as this novel care mode omits transportation and lost productivity costs [45-47], leaving only medical and labor costs. When comparing these 2 cost categories, our research showed that costs associated with virtual visits remained lower. This is consistent with previous research [48]. Our study also found that virtual visits are associated with lower medical costs. A possible explanation is that the frequency of prescribing laboratory tests was lower during telemedicine visits than during in-person visits [49]. Considering the benefits of telemedicine in terms of accessibility and affordability, virtual care will be a financially efficient method of delivering health care services in remote and impoverished regions with inadequate medical infrastructure, as well as in situations with restricted resources due to wars.

### Limitations

In this study, we showcased successful telemedicine implementation in China. By comprehensively examining 2 service models in the 2 cohorts, our research not only holds significant clinical implications but also provides strong evidence for the cost-effectiveness of telemedicine. However, it is important to acknowledge that our research possesses specific constraints. One constraint pertains to the use of a retrospective methodology, which is an observational approach that seeks to examine the association between occurrences using historical data. Its inherent nature makes it difficult to establish causal relationships [50]. To address the first problem, we used the PSM approach. PSM offers a solution to address selection bias by effectively managing confounding variables. Given the restricted sample size and data availability, our study used 7 variables pertaining to the patients’ characteristics, health-seeking behaviors, and costs in the PSM. Additional factors that may also contribute to the change in visit modality, such as socioeconomic status, digital literacy, and the willingness to adopt new technologies, could not be included. Nonetheless, PSM remains a significant means for probing the mechanisms through which telemedicine improves outcomes. In addition, the treatment course is a natural sequential process. Some patients opt to switch to approaches integrating telemedicine after in-person treatment. The evidence maximizes the strength of a causal relationship between telemedicine use and improved treatment outcomes as well as changes in health-seeking behaviors. 

Another issue is that some data could not be collected since the patients had finished their treatment course prior to our study. As a result, there were 2 repercussions. The first was our inability to evaluate the factors contributing to patient conversion from virtual to in-person appointments. A previous study found that 2% of individuals went back to physical visits because of language barriers [51]. Although we lacked the necessary data for a comparative analysis, we may deduce that situations in which virtual and in-person visits occur on the same day may involve certain needs that cannot be effectively addressed remotely. Only 1 patient had an in-person visit 1 hour and 22 minutes after having a virtual visit. Although the patient conversion rate we observed is comparable to that in prior research, additional research is necessary to elucidate patients’ inclinations to modify their health-seeking behaviors. The second repercussion was that we had to estimate transport costs and lost productivity costs using the patients’ demographic information and previously published work [31].

Last, we could not measure patient satisfaction data in this work. For in-person visits, given that the satisfaction was collected anonymously, it was challenging to correlate satisfaction ratings with the patients included in this study. For virtual visits, despite the fact that patients had to reveal their real names, the rate of satisfaction reports was merely 3.8%. With only 107 virtual visits, the TIC group only had 4 reviews, an inadequate sample size for further research. Although this issue may impact optimization of the 2 service models in the future, it does not impact the conclusion of this paper.

### Conclusions

Our study reveals a possible mechanism for how care integrating telemedicine could lead to improved clinical outcomes compared with in-person care. Telemedicine serves as a viable alternative in situations where access is restricted, without causing abuse of medical resources in terms of the visit frequency and average cost. Our research underscores the transformative potential of telemedicine integrated into care for addressing health care challenges, exemplified by the enhanced efficacy observed in managing short stature. As we forge ahead, the intersection of big data, large language models, and digital medicine holds immense promise for shaping the future landscape of health care delivery. The integration of these cutting-edge technologies offers exciting avenues for personalized, efficient, and accessible health care solutions, paving the way for novel research directions that can revolutionize patient outcomes and propel the field toward unprecedented advancements.

## Appendix

Multimedia Appendix 1 STROBE checklist.

## Abbreviations

25-OH VitD3: 25-hydroxyvitamin D3
EMR: electronic medical record
FT4: free thyroxine
IGF-1: insulin-like growth factor 1
IPC: in-person care
PSM: propensity score matching
TIC: telemedicine integrated care
TSH: thyroid-stimulating hormone
WHO: World Health Organization

## Data Availability

The data sets used in this study are available from the corresponding author upon reasonable request.
